# Note on Examination of Milk for Tubercle Bacilli

**Published:** 1900-07

**Authors:** E. W. Hammond

**Affiliations:** Agricultural College, Guelph, Ontario


### NOTE ON EXAMINATION OF MILK FOR TUBERCLE BACILLI.

By E. W. Hammond, D.V.S.,
AGRICULTURAL COLLEGE, GUELPH, ONTARIO.

After having tried various methods for the detection of tubercle bacilli in suspected milks, I can recommend the following as being that which in my hands has given me most satisfactory results.

The milk to be examined should be collected fresh in sterilized bottles, preferably those holding about eight ounces, and these bottles should be closed, not by cork, but by a tampon of (non-absorbent) cotton-wool. Such milk can be examined immediately, or if it be kept for a time 2½ per cent. glacial carbolic acid should be added as a preservative, and the bottle be put away in a cool, dark cupboard.

In either case, whether employing the fresh or the carbolized milk, an equal quantity of water is added to the milk, which is then shaken briskly, and samples are taken and centrifugalized. My usual procedure is to take another bottle of the same size and divide the milk between the two, adding to each the equal amount of water.

It is difficult to make any statement as to how long centrifugalization should occur, different workers employing different forms which rotate at very various speeds. Using an electrical centrifuge, I generally leave the tubes of milk for at least half an hour.

After centrifugalization the sediment is removed with a fine pipette, and a drop of this is placed on a clean cover-glass, formed into a film, dried, and then stained by the ordinary method (carbol fuchsin, followed by Gabbet's blue). To obtain good results I have found that the method of floating the cover-slip "butter-side down" on the surface of heated carbolic fuchsin in a water-glass is far preferable to the method of pouring the fuchsin on to the surface of the cover-slip and then boiling over a flame.

I may add that after this dilution of the milk with an equal quantity of water so little fat material comes down in the sediment that it is unnecessary to use ether or other reagent to dissolve off the fat. Where the cream and surface layers after centrifugalization are examined for the bacilli it is necessary to dissolve off the fats. Using this method it is my experience that it is a simpler and surer method to examine the sediment.

